# Comparison of a *Pf*HRP2-based rapid diagnostic test and PCR for malaria in a low prevalence setting in rural southern Zambia: implications for elimination

**DOI:** 10.1186/s12936-015-0544-3

**Published:** 2015-01-28

**Authors:** Natasha M Laban, Tamaki Kobayashi, Harry Hamapumbu, David Sullivan, Sungano Mharakurwa, Philip E Thuma, Clive J Shiff, William J Moss, for Southern Africa International Centers of Excellence for Malaria Research

**Affiliations:** Macha Research Trust, Choma, Zambia; Department of Epidemiology, Johns Hopkins Bloomberg School of Public Health, Baltimore, MD USA; W. Harry Feinstone Department of Molecular Microbiology and Immunology, Johns Hopkins Bloomberg School of Public Health, Baltimore, MD USA

**Keywords:** *Pf*HRP2-RDT, Nested PCR, Quantitative PCR, Malaria, Elimination, Zambia

## Abstract

**Background:**

Rapid diagnostic tests (RDTs) detecting histidine-rich protein 2 (*Pf*HRP2) antigen are used to identify individuals with *Plasmodium falciparum* infection even in low transmission settings seeking to achieve elimination. However, these RDTs lack sensitivity to detect low-density infections, produce false negatives for *P. falciparum* strains lacking *pfhrp2* gene and do not detect species other than *P. falciparum*.

**Methods:**

Results of a *Pf*HRP2-based RDT and *Plasmodium* nested PCR were compared in a region of declining malaria transmission in southern Zambia using samples from community-based, cross-sectional surveys from 2008 to 2012. Participants were tested with a *Pf*HRP2-based RDT and a finger prick blood sample was spotted onto filter paper for PCR analysis and used to prepare blood smears for microscopy. Species-specific, real-time, quantitative PCR (q-PCR) was performed on samples that tested positive either by microscopy, RDT or nested PCR.

**Results:**

Of 3,292 total participants enrolled, 12 (0.4%) tested positive by microscopy and 42 (1.3%) by RDT. Of 3,213 (98%) samples tested by nested PCR, 57 (1.8%) were positive, resulting in 87 participants positive by at least one of the three tests. Of these, 61 tested positive for *P. falciparum* by q-PCR with copy numbers ≤ 2 x 10^3^ copies/μL, 5 were positive for both *P. falciparum* and *Plasmodium malariae* and 2 were positive for *P. malariae* alone. RDT detected 32 (53%) of *P. falciparum* positives, failing to detect three of the dual infections with *P. malariae*. Among 2,975 participants enrolled during a low transmission period between 2009 and 2012, sensitivity of the *Pf*HRP2-based RDT compared to nested PCR was only 17%, with specificity of >99%. The *pfhrp* gene was detected in 80% of *P. falciparum* positives; however, comparison of copy number between RDT negative and RDT positive samples suggested that RDT negatives resulted from low parasitaemia and not *pfhrp2* gene deletion.

**Conclusions:**

Low-density *P. falciparum* infections not identified by currently used *Pf*HRP2-based RDTs and the inability to detect non-*falciparum* malaria will hinder progress to further reduce malaria in low transmission settings of Zambia. More sensitive and specific diagnostic tests will likely be necessary to identify parasite reservoirs and achieve malaria elimination.

**Electronic supplementary material:**

The online version of this article (doi:10.1186/s12936-015-0544-3) contains supplementary material, which is available to authorized users.

## Background

A substantial reduction in the burden of malaria has been achieved in several countries of sub-Saharan Africa [1]. Sustaining this level of malaria control and progressing towards elimination will depend on sensitive and specific diagnostic tools to identify persistent reservoirs of infection. Although microscopy remains the diagnostic gold standard, malaria rapid diagnostic tests (RDTs) are widely used throughout malaria endemic areas, particularly in rural settings where access to health facilities with trained microscopists is limited. As transmission declines, detection of infected individuals is critical to achieving and sustaining control. Evaluation of RDT performance in regions of low transmission is necessary.

Zambia has recorded a significant decrease in parasite prevalence as documented by malaria indicator surveys, with parasite prevalence among children younger than five years of age decreasing from 21.8% in 2006 to 14.9% in 2012 [2]. Southern Province, Zambia reported a parasite prevalence of less than 10% and is considered a potential area for malaria elimination. RDTs based on detecting histidine-rich protein 2 (*Pf*HRP2), an antigen produced only by *Plasmodium falciparum,* have been used in Zambia since their national introduction in 2009 [3]. However, RDTs lack the sensitivity to detect low-density infections compared to more sensitive molecular methods such as polymerase chain reaction (PCR) [4-6]. Furthermore, *P. falciparum* parasites with a deletion of the *pfhrp2* gene can cause patent bloodstream infection but false negative RDT results [7-11]. Identification of other human malaria parasite species is not possible with *Pf*HRP2-based RDTs and non-*falciparum* malaria may become more apparent as transmission decreases. To assess the validity of *Pf*HRP2-based RDTs in a hypoendemic area moving towards elimination, results of a *Pf*HRP2-based RDT were compared to those of a *Plasmodium* nested PCR. Using real-time, quantitative PCR (q-PCR), identification of parasite species, quantification of parasitaemia and potential *pfhrp* gene deletions were assessed. This is the first study to examine the presence of non-*P.falciparum* species and *pfhrp* gene deletion in southern Zambia and the findings have implications for RDT-based strategies to achieve malaria elimination in this region.

## Methods

The study consisted of community-based, cross-sectional surveys conducted in the catchment area of Macha Hospital located in Choma District, Southern Province, Zambia between 2008 and 2012. Macha Hospital is located approximately 70 km from the nearest town of Choma on a plateau at an altitude of approximately 1,100 metres above sea level and in a habitat characterised as Miombo woodland. There is a single rainy season from approximately November through April, followed by a cool, dry season from April to August and a hot, dry season from August to November. The catchment area is populated by traditional villagers living in small scattered homesteads. *Anopheles arabiensis* is the primary vector responsible for malaria transmission [12], which peaks during the rainy season. The Southern Province of Zambia historically had hyperendemic *P. falciparum* transmission but the parasite prevalence and number of hospitalizations for malaria declined dramatically over the past decade [13].

Cross-sectional surveys were conducted every other month beginning February 2008. Household and participant selection was as described previously [14], but in brief, households were randomly selected from satellite images and all household residents were eligible to participate. Informed consent was obtained from participating adults and from parents or guardians of children younger than 16 years. Questionnaires were administered to collect information on age, sex and history of recent malaria and treatment. Finger prick blood samples were used to prepare thick and thin blood smears for microscopy, RDT (ICT Diagnostics, Cape Town, South Africa) and spotted onto filter paper (Whatman 903™ Protein Saver card) as dried blood spots (DBS). The ICT Diagnostics RDT detects *Pf*HRP2 antigen. Blood smear and DBS samples were transported to a molecular laboratory at Macha Research Trust, located within the study area, where both microscopy and *Plasmodium* nested PCR analysis were performed. Storage of DBS was at −20°C, with each filter paper card individually sealed in a plastic bag containing desiccant prior to nucleic acid extraction and PCR analysis. DBS collected from February to September 2008 were initially stored at room temperature (within individually sealed plastic bags with desiccant) but subsequently stored at −20°C.

Microscopic examination was performed at the Macha Research Trust laboratory within approximately three days of sample collection. Thin blood smears were air-dried, fixed with methanol and Giemsa-stained whereas thick blood smears were air dried and then Giemsa-stained without fixing with methanol. Parasitological diagnosis was made independently by two microscopists, with discrepancies resolved by a third reader. Parasite count was recorded per 1,000 white blood cells.

All laboratory assays except q-PCR were performed at the Macha Research Trust laboratory in Zambia. A Chelex© extraction method was used to recover parasite DNA from dried blood spots [15] within approximately one year of sample collection for DBS collected from 2010 to 2012 and approximately three to five years for DBS collected in 2008 and 2009. Positive and negative control samples spotted as dried blood on filter paper were included in each extraction experiment. Positive controls consisted of parasitized blood from laboratory cultures at 1,000 parasites/μL. Negative controls consisted of blood from individuals with no travel history to malaria endemic areas. The dried blood spots were placed in 1.5 mL microcentrifuge tubes, 1 mL of 0.1% weight by volume saponin in 1x phosphate buffered saline (PBS) was added and the mixture was incubated for 10 minutes at room temperature. The tubes were centrifuged for two minutes at 14,000 rpm, the supernatant discarded and 1 mL of 1 × PBS was added. The tubes were again centrifuged for 2 minutes at 14,000 rpm, the supernatant discarded and 150 μL of 2% weight by volume Chelex© solution and 50 μl of DNase free water were added and the tubes boiled for 8 minutes. The tubes were then centrifuged for one minute at 14,000 rpm and approximately 150 μL of DNA was stored at −20°C.

A *Plasmodium* nested PCR assay was performed within approximately one month following the DNA extraction for detection of asexual stage parasite DNA using two sets of primers targeting a segment of the mitochondrial cytochrome b gene (*cytb*) conserved in the four major human *Plasmodium* parasites [16]. In the primary PCR step, 6 μL of DNA extract was pipetted into 0.2 mL tubes containing a 19 μL reaction mix made up of DNase free water and final concentrations of dNTPs, 10× PCR buffer, magnesium chloride, forward and reverse primers and DNA Taq polymerase as 0.2 mΜ, 1×, 3 mΜ, 1 μΜ and 2 units in 25 μL reaction mix, respectively. In the nested PCR step, 3 μL of the primary PCR product was added to 0.2 mL PCR tubes containing 22 μL of reaction mix containing DNase free water and final concentrations of dNTPs, 10× buffer, magnesium chloride, forward and reverse primers and Taq DNA polymerase as 0.2 mΜ, 1×, 2.5 mΜ, 1 μΜ and 2 units in 25 μL reaction mix, respectively. No-template controls were included in each experiment and reactions were run in a Techne™ TC-412 thermo cycler (See Additional file 1). Amplified product was detected by electrophoresis on 1% agarose gel and viewed under UV light as an 815 base pair DNA band.

DNA extracts from individuals positive by either microscopy, RDT or nested PCR were shipped with ice packs to the United States and stored at −20°C prior to further analyses by q-PCR. *P. falciparum*, *P. malariae* and *pfhrp* gene detection by q-PCR using specific primer sets was performed at the Johns Hopkins Bloomberg School of Public Health between July and October 2013. Amplification was demonstrated to require ten-fold dilution with nuclease free water to minimize inhibition of the q-PCR reaction due to substrate in the crude Chelex DNA extract. Primers for species-specific *cytb* were used to detect *P. falciparum* and *P. malariae*. The primers used for the detection of the *pfhrp* gene were designed to amplify the highly conserved secretary leader of *pfhrp* as previously reported [17]. The primer was designed to confirm the presence of *pfhrp2* or *pfhrp3* in a single reaction, with expected product sizes of 278 base pairs and 259 base pairs for *pfhrp2* and *pfhrp3*, respectively (See Additional file 1). Five μL of reaction mix containing 5 μL of 2x iQ™ SYBR® Green supermix (Bio-Rad) and 400 nΜ primer were added in duplicate to a 384-well plate along with 5 μL of ten-fold diluted DNA extract. Standards were generated by serial dilution of genomic DNA. Ten-fold dilutions of laboratory-cultured 3D7 genomic DNA were used as standards for *P. falciparum* and *pfhrp* gene detection (See Additional file 2), whereas *P. malariae* genomic DNA from a single infected individual was used as a standard for detection of *P. malariae.* The loaded plates were centrifuged for 5 minutes at 2,000 rpm and reactions were run in a Bio-Rad CFX384™ real time thermo cycler. Baseline thresholds were uniform for all experiments of the same assay and cycle count values falling within the range set by the standards were used to determine positive results for each q-PCR assay. Absolute quantification was used to determine gene copy numbers for the *P. falciparum* and *pfhrp* q-PCR assays based on the standard curve generated from the 3D7 dilutions (See Additional file 3).

## Results

A total of 3,292 study participants were enrolled from 2008 through 2012 (See Additional file 4). Of these 3,292 participants, 12 (0.4%) were positive by microscopy and 42 (1.3%) were positive by *Pf*HRP2-based RDT. Nested PCR was performed on 3,213 (98%) samples and 57 (1.8%) positive individuals were identified. In 2008, nested PCR was performed on 276 (87%) of the 317 samples collected; however, the prevalence of nested PCR positivity (3.6%) was lower than RDT positivity (10%) presumably as a consequence of long storage time. Hence, results from 2008 were not included in the comparisons between RDT and nested PCR as potential nested PCR positive but RDT and microscopy negative samples may have been missed. Consequently, 2,975 samples were used for comparison of RDT and nested PCR for the study period 2009 to 2012.

### Parasite prevalence by microscopy, RDT and nested PCR from 2009 to 2012

**Figure 1:**
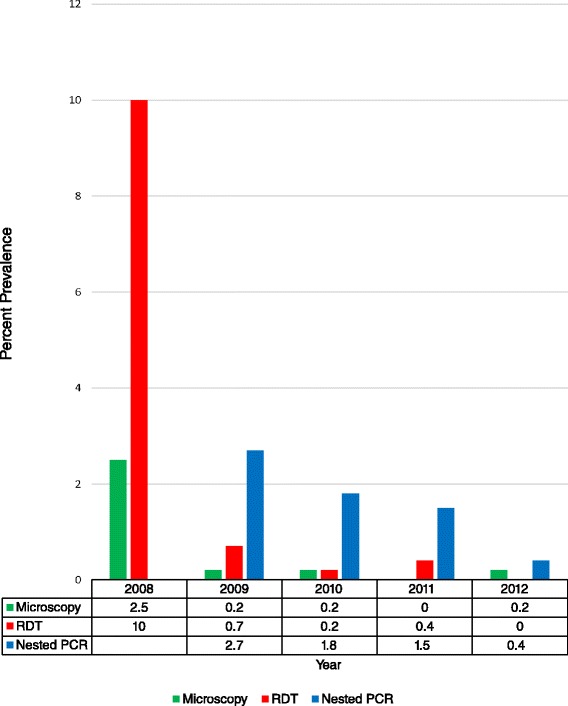
**Parasite prevalence as measured by microscopy,** ***Pf*** **HRP2-RDT and** ***Plasmodium*** **nested PCR.** Prevalence by microscopy and ICT Diagnostics RDT per year on a total of 3, 292 participants and by nested PCR on 3,213 (98%) participants enrolled between 2008 and 2012. *PCR data for 2008 excluded.

Of the 2,975 participants enrolled in the cross sectional surveys during the four-year period of low transmission from 2009 through 2012, only four individuals were positive for malaria by microscopy (0.13%) and ten by RDT (0.34%). Of the 2,937 (99%) samples tested by nested PCR, 47 were positive (1.6%). The parasite prevalence by RDT was 0.74% in 2009, 0.23% in 2010, 0.4% in 2011 and 0% in 2012, whereas the parasite prevalence by nested PCR was 2.7% in 2009, 1.8% in 2010, 1.5% in 2011 and 0.44% in 2012 (Figure 1). The sensitivity of the *Pf*HRP2-based RDT compared to the nested PCR was only 17% (Table 1). In this study setting in which all household residents were tested regardless of symptoms, in contrast to a clinical setting where the prevalence of malaria would be higher, the positive predictive value of the RDT compared to nested PCR was 80%, with a negative predictive value of 99%.

**Table 1:** **Comparison of** ***Pf*** **HRP2-based RDT results with microscopy and nested PCR from 2009 – 2012**

	**Microscopy positive**	**Microscopy negative**	**Total**	**PCR positive**	**PCR negative**	**Total**
**RDT positive**	2	8	10	8	2	10
**RDT negative**	2	3280	3282	39	2888	2927
**Total**	4	3288	3292	47	2890	2937*

*Of the 2,975 enrolled during this period, 2,937 (99%) were tested by both RDT and nested PCR.

Sensitivity of *Pf*HRP2-based RDT compared to microscopy = 0.50.

Specificity of *Pf*HRP2-based RDT compared to microscopy = > 0.99.

Positive predictive value of *Pf*HRP2-based RDT compared to microscopy = 20%.

Negative predictive value of *Pf*HRP2-based RDT compared to microscopy = > 99%.

Sensitivity of *Pf*HRP2-based RDT compared to PCR = 0.17.

Specificity of *Pf*HRP2-based RDT compared to PCR = > 0.99.

Positive predictive value of *Pf*HRP2-based RDT compared to PCR = 80%.

Negative predictive value of *Pf*HRP2-based RDT compared to PCR = 99%.

### Identification of *Plasmodium* species

The q-PCR for *P. falciparum* and *P. malariae* was performed on 49 samples collected from 2009 through 2012 that were positive for malaria by RDT, microscopy or nested PCR as well as an additional 38 samples collected during 2008 and positive by at least one of the three tests, yielding a total of 87 samples on which q-PCR was performed.

Of these 87 samples, 56 (64%) were positive by q-PCR for *P. falciparum* alone, five were positive for both *P. falciparum* and *P. malariae* and two were positive for *P. malariae* alone (Table 2). Thus, 61 (70%) were positive for *P. falciparum* and seven for *P. malariae* (0.2% of all participants). The q-PCR was negative for 24 individuals who tested positive by either microscopy, RDT or nested PCR. Of these 24, one was positive by microscopy only, eight were positive by RDT only, thirteen were positive by nested PCR only, and two were positive by both RDT and nested PCR.

**Table 2:** ***Plasmodium*** **species identification by q-PCR**

	***P. falciparum***	***P. malariae***	***P. falciparum*** **and** ***P. malariae***
Total positive by q-PCR*	**56**	**2**	**5**
Also positive by microscopy	10	0	1
Also positive by RDT	30	0	2
Also positive by nested PCR	37	2	3

*Of the 87 tested samples that were positive by RDT, microscopy or PCR, 24 were negative by q-PCR.

Of the 42 individuals positive by RDT from 2008 through 2012, 32 were positive for *P. falciparum* by q-PCR. Eight of the 10 individuals positive by RDT but negative by q-PCR were also negative by microscopy and nested PCR, suggesting these individuals had persistent *Pf*HRP2 antigenaemia in the absence of visible parasites or parasite DNA. Only one of these eight individuals reported prior treatment, but with an herbal medicine and not artemisinin combination therapy. The remaining two samples were positive by nested PCR, which suggested either false positive results for both RDT and nested PCR or false negative q-PCR. Of the 12 individuals positive by microscopy, only one was negative by q-PCR, suggestive of either false positive microscopy or false negative q-PCR.

### Quantification of *P. falciparum* parasitaemia

The *P. falciparum* genome size is 23 mega bases [18] and on the assumption that the average weight of one mole of a base pair is 650 g, 1 ng of parasite genomic DNA corresponds to 40,000 copies of the target gene. The limit of detection for *P. falciparum* q-PCR was determined to be 0.04 copies/μL. The *cytb* gene copy numbers quantified by q-PCR for the 61 samples positive for *P. falciparum* ranged from 1 × 10^−1^ copies/μL to 2 × 10^3^ copies/μL. The mean copy number for individuals positive by both RDT and *P. falciparum cytb* q-PCR was 97 copies/μL (range 0.05 to 2178 copies/μL, SD = 381) in contrast to a mean copy number of 8 copies/μL (range 0.1 to 96 copies/μL, SD = 18) for individuals who were RDT negative but *P. falciparum* q-PCR positive (Table 3).

**Table 3:** ***Pfcytb*** **and** ***Pfhrp*** **copy numbers in individuals positive for** ***P. falciparum*** **by q-PCR**

	***P. falciparum*** **q-PCR positive**	***Pfhrp*** **q-PCR result (N)**	***Pfcytb*** **mean copy number/μL**	***Pfhrp*** **mean copy number/μL**
**RDT positive**	32	positive (26)	97	1827
		negative (6)		NA
**RDT negative**	29	positive (23)	8	237
		negative (6)		NA

NA = not amplified.

### Detection of *pfhrp* gene in *P. falciparum* infections

The limit of detection for the *pfhrp* gene by q-PCR was 4 copies/μL. The *pfhrp* gene was present in 49 (80%) of the 61 samples confirmed to have *P. falciparum* DNA by q-PCR. Of the 32 samples positive by both RDT and q-PCR, the *pfhrp* gene was detectable in 26 (81%). Of the 29 RDT-negative but q-PCR positive samples for *P. falciparum*, the *pfhrp* gene was detected in 23 (79%). The mean copy number of *Pfcytb* q-PCR was approximately 13-fold lower among RDT negative samples compared to RDT positive samples. Similarly, the mean *pfhrp* copy number was approximately 7-fold lower among RDT negative samples compared to RDT positive samples (Table 3).

## Discussion

In a region of declining malaria transmission in southern Zambia, a *Pf*HRP2-based RDT had low sensitivity in detecting *P. falciparum* infections and failed to detect a small number of mixed infections with *P. malariae*. Failure to detect the *pfhrp* gene in approximately 20% of infections with *P. falciparum* was likely a consequence of low parasite density rather than deletion of the *pfhrp* gene. Although the absolute prevalence of malaria was low, PCR detected four to nine times the number of infections than the *Pf*HRP2-based RDT.

Field evaluations of *Pf*HRP2-based RDTs have reported high sensitivities in medium to high malaria transmission settings [19,20] where parasite densities commonly exceed 200 parasite/μL. However, recent studies conducted in pre-elimination settings in sub-Saharan Africa have shown a reduction in sensitivity of RDTs for malaria detection. The sensitivity of RDT compared to PCR in Zanzibar was 76.5% [21], higher than observed in Zambia. In regions of declining transmission, this may reflect low-level parasitaemia in individuals with clinical immunity acquired when transmission was higher. Incorporation of more sensitive diagnostics may be necessary in such settings to eliminate the residual infectious reservoir, consistent with recent recommendations by the WHO Malaria Policy Advisory Committee on the role of nucleic acid-based malaria diagnosis in low transmission settings [22]. If transmission remains low for an extended period, clinical immunity may be lost, resulting in more frequent symptomatic infections with higher levels of parasitaemia and increased sensitivity of RDTs.

Non-*falciparum Plasmodium* species have previously been described in Zambia although they constitute a small proportion of infections. Infection with *Plasmodium ovale* was described in the 1960’s [23] and infection with *P. malariae* was estimated to comprise 2-4% of malaria infections in Zambia. In 2012, the prevalence of infection with *P. malariae* was 2.1% in Nchelenge District in northern Zambia [24].

Although *P. falciparum* not expressing *Pf*HRP2 has been reported from some regions [25], the *pfhrp2* gene was present in the majority of the circulating *P. falciparum* parasites in southern Zambia. This is the first report of prevalence of *pfhrp2* in circulating *P. falciparum* parasites in Zambia and continued monitoring for the occurrence of *pfhrp* gene deletions will be necessary as *Pf*HRP2-based RDTs continue to be used.

## Conclusions

Current efforts to achieve malaria elimination in southern Zambia using reactive case detection based on RDTs may be insufficiently sensitive to interrupt transmission. Alternative strategies, such as focal drug administration or the use of more sensitive and specific diagnostic tests, may be necessary to eliminate the parasite reservoir.

## Additional files

Additional file 1:
**Primer sequences and reaction conditions for PCR assays.** Amplification of asexual-stage *Plasmodium* parasite used primers targeting a region of the mitochondrial cytochrome b (*cytb*) gene conserved in the four major human malaria parasites. Identification of species and detection of *pfhrp* gene were based on species-specific *cytb* primers and primers amplifying a highly conserved secretary leader of the *pfhrp* gene respectively. Additional file 2:
**Amplification curve for** ***Plasmodium falciparum*** **q-PCR.** Q-PCR amplification curve for *P. falciparum* detection was generated by Bio-Rad CFX-384 Thermocycler using ten-fold dilutions of 3D7 genomic DNA from a 1 nanogram stock. Additional file 3:
**Standard curve for absolute quantification of** ***Plasmodium falciparum*** **parasitaemia.** Quantification was based on 1 nanogram of *P. falciparum* genomic DNA corresponding to 40,000 target gene copy number. Additional file 4:
**Number of samples tested by Microscopy, RDT, nested PCR and the q-PCR per study year.**
