# Supplementary figures and images for: Comparison of a PfHRP2-based rapid diagnostic test and PCR for malaria in a low prevalence setting in rural southern Zambia: implications for elimination
Source: Malar J. 2015 Jan 28;14:25. doi: 10.1186/s12936-015-0544-3 (PMC4340619; doi:10.1186/s12936-015-0544-3)

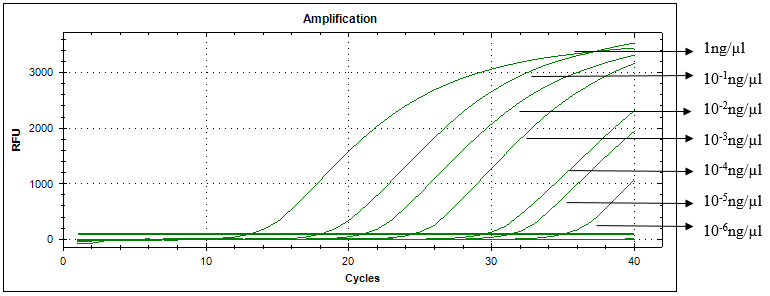

Supplement: Additional file 2: — Amplification curve for Plasmodium falciparum q-PCR. Q-PCR amplification curve for P. falciparum detection was generated by Bio-Rad CFX-384 Thermocycler using ten-fold dilutions of 3D7 genomic DNA from a 1 nanogram stock. [file 12936_2015_544_MOESM2_ESM.png]

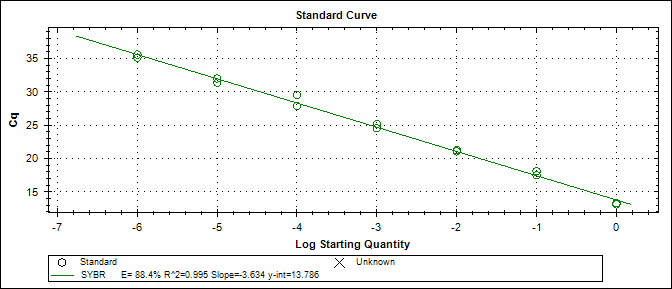

Supplement: Additional file 3: — Standard curve for absolute quantification of Plasmodium falciparum parasitaemia. Quantification was based on 1 nanogram of P. falciparum genomic DNA corresponding to 40,000 target gene copy number. [file 12936_2015_544_MOESM3_ESM.png]
