# Supplementary material for: Comparison of a PfHRP2-based rapid diagnostic test and PCR for malaria in a low prevalence setting in rural southern Zambia: implications for elimination
Source: Malar J. 2015 Jan 28;14:25. doi: 10.1186/s12936-015-0544-3 (PMC4340619; doi:10.1186/s12936-015-0544-3)
Supplement: Additional file 4: — Number of samples tested by Microscopy, RDT, nested PCR and the q-PCR per study year. [file 12936_2015_544_MOESM4_ESM.pdf]

Year	Number of samples tested by			Positive by at least one test
	**Microscopy**	**RDT**	**Nested PCR**	
2008	317	317	276	38
2009	676	676	638	18
2010	871	871	871	16
2011	740	740	740	12
2012	688	688	688	3
**Total**	3292	3292	3213	87*

*Samples positive by at least one of the three tests were used for the q-PCR analysis
